# Total laparoscopic vs. open liver resection: comparative study with propensity score matching analysis

**DOI:** 10.1590/0102-672020190001e1494

**Published:** 2020-05-18

**Authors:** Bruno Silva de ASSIS, Fabricio Ferreira COELHO, Vagner Birk JEISMANN, Jaime Arthur Pirola KRUGER, Gilton Marques FONSECA, Ivan CECCONELLO, Paulo HERMAN

**Affiliations:** 1 Postgraduate Course in Digestive Surgery, Colégio Brasileiro de Cirurgia Digestiva (CBCD), São Paulo, Brazil; 2 Digestive Surgery Division, Department of Gastroenterology, School of Medicine, Universityof São Paulo, São Paulo, Brazil; 3 Cancer Institute of São Paulo State (ICESP), São Paulo, Brazil

**Keywords:** Hepatectomy, Laparoscopy, Hepatic neoplasms/surgery, Comparative study, Propensity score, Hepatectomia, Laparoscopia, Neoplasias hepáticas/cirurgia, Estudo comparativo, Pontuação de propensão

## Abstract

***Background*:**

There have been an increasing number of articles that demonstrate the potential benefits of minimally invasive liver surgery in recent years. Most of the available evidence, however, comes from retrospective observational studies susceptible to bias, especially selection bias. In addition, in many series, several modalities of minimally invasive surgery are included in the same comparison group.

***Aim*:**

To compare the perioperative results (up to 90 days) of patients submitted to total laparoscopic liver resection with those submitted to open liver resection, matched by propensity score matching (PSM).

***Method*:**

Consecutive adult patients submitted to liver resection were included. PSM model was constructed using the following variables: age, gender, diagnosis (benign vs. malignant), type of hepatectomy (minor vs. major), and presence of cirrhosis. After matching, the groups were redefined on a 1:1 ratio, by the nearest method.

***Results*:**

After matching, 120 patients were included in each group. Those undergoing total laparoscopic surgery had shorter operative time (286.8±133.4 vs. 352.4±141.5 minutes, p<0.001), shorter ICU stay (1.9±1.2 vs. 2.5±2.2days, p=0.031), shorter hospital stay (5.8±3.9 vs. 9.9±9.3 days, p<0.001) and a 45% reduction in perioperative complications (19.2 vs. 35%, p=0.008).

***Conclusion*:**

Total laparoscopic liver resections are safe, feasible and associated with shorter operative time, shorter ICU and hospital stay, and lower rate of perioperative complications.

## Graphical Abstract

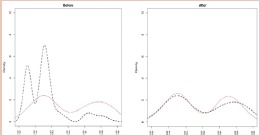

## RESUMO

***Racional*:**

Com a disseminação da cirurgia hepática minimamente invasiva tem-se observado nos últimos anos número crescente de trabalhos que demonstram seus potencias benefícios. No entanto, a maior parte da evidência disponível provém de estudos observacionais retrospectivos sujeitos a vieses, em especial, os de seleção. Além disso, em muitas casuísticas são incluídas no mesmo grupo diversas modalidades de operações minimamente invasivas.

***Objetivo*:**

Comparar os resultados perioperatórios (até 90 dias) de pacientes submetidos a ressecções hepáticas totalmente laparoscópicas com pacientes contemporâneos por cirurgias abertas, pareados por pontuação de propensão (propensity score matching PSM), submetidos a ressecções hepáticas convencionais.

***Método*:**

Foram estudados pacientes adultos consecutivos submetidos à ressecção hepática. Para homogeneização dos grupos foi utilizado pareamento por pontuação de propensão, utilizando a variável idade, gênero, tipo de doença (benigna vs. maligna), tipo de hepatectomia (maior vs. menor) e presença de cirrose. A partir disto, os grupos foram redefinidos com proporção 1:1, pelo método nearest.

Após o pareamento foram incluídos 120 pacientes em cada grupo. Os submetidos à operação totalmente laparoscópica apresentaram menor tempo cirúrgico (286,8±133,4 vs. 352,4±141,5 min, p<0,001), menor tempo de internação em unidade de terapia intensiva (1,9±1,2 vs. 2,5±2,2dias, p=0,031), menor tempo de internação hospitalar (5,8±3,9 vs. 9,9±9,3dias, p<0,001) e redução de 45% nas complicações perioperatórias (19,2 vs. 35%, p=0,008).

***Conclusão*:**

As ressecções hepáticas totalmente laparoscópicas são exequíveis, seguras e associadas à menor tempo operatório, menor tempo de internação em unidade de terapia intensiva e internação hospitalar, além de diminuição nas complicações perioperatórias.

## Graphical Abstract

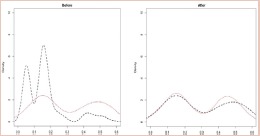

## INTRODUCTION

Laparoscopic liver resections (LLR) are complex procedures demanding long learning curve, requiring experienced liver surgeons with training in advanced laparoscopy^8^ ^,^ ^23^. However, these procedures have become increasingly common in recent years, driven by the good initial results that demonstrate their safety, feasibility, and potential benefits over the open liver resections (OLR)^13^ ^,^ ^18^ ^,^ ^26^.

The best candidates for LLR are those with lesions located in the anterolateral segments of the liver (segments 2, 3, 4b, 5 and 6), also known as “laparoscopic segments”^5^ ^,^ ^8^. Currently, laparoscopic minor resections in these segments and left lateral sectionectomy have been considered the gold standard approach in specialized centres^5^ ^,^ ^19^ ^,^ ^28^ **.** Resection of bilateral lesions, nodules in posterosuperior segments or in central locations in the liver (segments 1, 4a, 7 and 8), and major hepatectomies (resection of ≥3 contiguous segments) are still challenging^9^ ^,^ ^10^ ^,^ ^14^. However, with the increase experience and development of alternative modalities of minimally invasive liver surgery (MILS) the technical difficulties could be overcome, allowing successful major LLR, such as left and right hepatectomies^21^. Recently, minimally invasive surgery has also been used for living donation^4^.

The most commonly minimally invasive modalities employed are totally laparoscopic (TLLR), hand-assisted and laparoscopy-assisted (hybrid) surgery^5^ ^,^ ^28^. Totally laparoscopic is the preferred approach, in this modality the procedure is carried out through laparoscopy, with an auxiliary incision made at the end of the surgery to retrieve the surgical specimen. Hand-assisted and hybrid resections were developed in order to overcome some limitations of the TLLR, and therefore expand the indications of MILS^8^ ^,^ ^19^. These approaches are especially useful in complex resections and centres in the early experience with MILS^7^ ^,^ ^9^ ^,^ ^15^.

Several studies have been published demonstrating potential benefits of MILS^6^ ^,^ ^18^. However, the available evidence is mostly based on retrospective observational studies, which are susceptible to bias, especially selection bias. OLR is more commonly indicated for patients with worse performance status and technical demanding resections^9^ ^,^ ^20^. 

Furthermore, in many studies different modalities of MILS are included in the same comparison group^6^. There are few studies that evaluate specific modalities of MILS^1^ ^,^ ^9^ ^,^ ^18^. Only recently randomized controlled trials and observational studies with the methodological concern of sample matching were published comparing the results of MILS and OLR^1^ ^,^ ^9^ ^,^ ^12^ ^,^ ^29^. 

The aim of this study was to compare the perioperative results (up to 90 days) of patients undergoing TLLR with contemporary patients undergoing OLR, paired by propensity score matching (PSM).

## METHODS

The Institutional Ethics Committee approved this research protocol. This study was conducted following STROBE (Strengthening the Reporting of Observational studies in Epidemiology) recommendations^27^. 

From a prospective database consecutive adults patients submitted to OLR and TLLR for primary and secondary lesions between June 2008 and January 2016 were evaluated. The exclusion criteria were patients submitted to two-stage hepatectomy or ALPPS (associating liver partition and portal vein ligation for staged hepatectomy); hilar cholangiocarcinoma; patients submitted to hand-assisted or hybrid resections; and patients with incomplete data. The indication of the surgical procedure was carried out after discussion in a multidisciplinary meeting.

Liver resections were defined according to Brisbane terminology^3^. Major hepatectomy was defined as resection of ≥3 segments. OLR was defined as those performed through incisions as: J-shape incision, “Chevron” or “Mercedes” incision. In TLLR, the entire procedure was performed by laparoscopy and an auxiliary incision was performed only for specimen retrieval (usually a Pfannenstiel incision).

The following preoperative characteristics were studied: age, gender, body mass index (BMI), preoperative laboratory test, American Society of Anesthesiologists (ASA) physical status score, preoperative diagnosis, size and location of the lesions, previous abdominal surgeries, presence of chronic liver disease and portal hypertension. Regarding intra and postoperative data: type of procedure, operative time, estimated blood loss, transfusion requirement, conversion rate, length of intensive care unit (ICU) stay and length of hospital stay, postoperative complications and mortality were evaluated.The specimens obtained were assessed for the frequency of free margins and smaller distance.

Postoperative morbidity was defined as any event occurring during the first 90 postoperative days and was stratified according to the Dindo-Clavien classification^11^. Postoperative biliary fistula was defined following the criteria proposed by the International Study Group of Liver Surgery^17^. Postoperative mortality was defined as death within 90 days after liver resection.

### Statistical analysis

**FIGURE 1:**
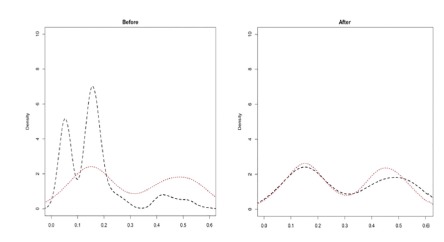
Density plots estimated for open (dashed line) and total laparoscopic (dotted line) resection groups before and after pairing by propensity score matching using the model with the variables age, gender, diagnosis (benign vs. malignant), type of hepatectomy (minor vs. major),and presence of cirrhosis.

Continuous data were expressed as median and interquartile range or mean and standard deviation (sd). Categorical variables were expressed as percentage. For comparison of means, the t-test was used when the distribution was normal. When data were not normally distributed, the non-parametric Mann-Whitney test or Brunner-Munzel T test was used. For the categorical variables, Fisher’s exact test or Chi-squared test was used. A p value<0.05 was considered statistically significant. PSM was used to avoid possible selection bias. The propensity score model was constructed using logistic regression including all variables collected, with a set of them being significant. After this, multivariate logistic regression was used, obtaining models including groups of variables. Using the Akaike information criterion method^16^, the model including the following variables: age, gender, diagnosis (benign vs. malignant), type of hepatectomy (minor vs. major), and presence of cirrhosis showed the best performance (Figure 1). From this, the comparison groups were redefined with a proportion of 1:1 through the nearest method.

## RESULTS

**FIGURE 2:**
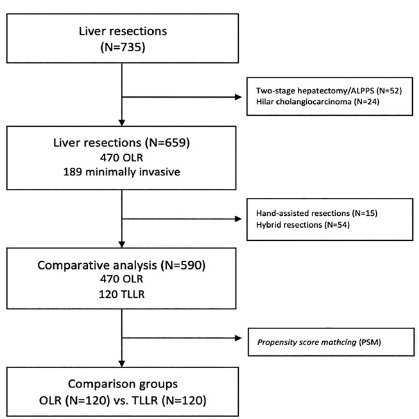
Flowchart of patients included in the study

During the study period 735 liver resections were performed. After applying the exclusion criteria, 590 were eligible for comparative analysis: 470 OLR and 120 TLLR. After match by PSM, 120 patients were included in each group (Figure 2).

Clinical and surgical characteristics of OLR and TLLR groups before and after matching are shown in Table 1. Before matching, the groups were not homogenous with a predominance of malignant diseases (75.5% vs. 63.3%, p=0.01), fewer patients with cirrhosis (7.9% vs. 20.8%, p<0.001), higher mean number of resected nodules (2.5±3.0 vs. 1.4±2.0, p<0.001), more major hepatectomies (41.1% vs. 17.5%, p<0.001) and associated procedures (22% vs. 13.5%, p=0.041)in the OLR group. Lower serum albumin level was observed in OLR group, despite both groups having values within normal range. After matching, the groups became homogenous for all baseline characteristics, with the exception of the number of resected nodules despite reducing the mean difference (2.5±3.0 vs.1.9±2.0, p<0.001).

**TABLE 1:** Baseline characteristics before and after propensity score matching (PSM)

	TLLR (n=120)	OLR before PSM (n=470)	p	OLR after PSM (n=120)	p
Age (years, mean±sd)	53.4±16.4	57.6±12.7	0.097	55.7±15.3	0.312
Gender (%) Male Female	48 (40%) 72 (60%)	235 (50%) 235 (50%)	0.242	47 (39.2%) 73 (60.8%)	1
BMI (kg/m^2^, mean±sd)	26.3±4.6	26.2±4.8	0.485	26.0±5.2	0.69
Diagnosis (%) Benign Malignant	44 (36.7%) 76 (63.3%)	115 (24.5%) 355 (75.5%)	0.01	40 (33.3%) 80 (66.7%)	0.684
Cirrhosis (%)	25 (20.8%)	34 (7.9%)	<0.001	27 (22.5%)	0.875
Number of nodules (mean± sd)	1.4±2.0	2.5±3.0	<0.001	1.9±2.0	<0.001
Size of largest nodule (mm, mean ± sd)	44.5±29.9	48.8±38.0	0.701	49.1±35.4	0.653
ASA classification (%) I II III IV	33 (27.5%) 80 (66.7%) 7 (5.8%) 0	87 (18.5%) 337 (71.7%) 43 (9.15%) 3 (0.7%)	0.124	28 (23.3%) 78 (65%) 11 (9.2%) 3 (2.5%)	0.11
Haemoglobin (g/dL, mean± sd)	13.2±1.5	13.1±1.7	0.446	13.1±1.6	0.492
Platelet count (10^3^/mm^3^, mean±sd)	221,678± 92,460	213,705± 104,544	0.156	224,641± 112,811	0.792
Bilirubin (g/dl, mean±sd)	0.6±0.3	0.7±1.3	0.573	0.9±1.7	0.206
Albumin (g/dl, mean±sd)	4.5±2.3	4.1±0.5	0.004	4.2±0.5	0.061
INR (mean±sd)	1.1±0.1	1.0±0.2	0.474	1.1±0.3	0.5
Creatinine (mg/dl, mean±sd)	0.8±0.2	0.9±0.7	0.161	0.9±0.6	0.724
Type of resection (%) Bisegmentectomy2-3 Bisegmentectomy 6-7 Right hepatectomy Left hepatectomy Segmentectomy Wedge resections Other resections	37 (30.8%) 7 (5.8%) 19 (15.8%) 2 (1.67%) 11 (9.2%) 43 (35.8%) 1 (0.8%)	36 (7.7%) 18 (3.8%) 109 (23.4%) 68 (14.5%) 38 (8.1%) 150 (31.9%) 34 (7.2%)		17 (14.2%) 5 (4.2%) 16 (13.3%) 9 (7.5 %) 11 (9.1%) 48 (40%) 3 (2.5%)	
Major hepatectomy (%)	21 (17.5%)	193 (41.1%)	<0.001	26 (21.7%)	0.515
Associated procedures(%)	16 (13.5%)	103 (22.0%)	0.041	18 (15%)	0.853

OLR=open liver resection;TLLR=total laparoscopic liver resection;sd=standard deviation; ASA=American Society of Anaesthesiologists;BMI=body mass index; INR=international normalised ratio

ALPPS=Associating liver partition and portal vein ligation for staged hepatectomy; OLR=open liver resection;TLLR=total laparoscopic liver resection

Perioperative results are shown in Table 2. After matching, patients submitted to TLLR showed shorter operative time (286.8±133.4 min vs. 352.4±141.5 min, p<0.001); less ICU requirement and shorter ICU stay (1.9±1.2 days vs. 2.5±2.2 days, p=0.031). An average reduction of almost four days in the length of hospital stay (5.8±3.9 days vs. 9.9±9.3 days, p<0.001) was observed. Additionally, we found a significant reduction (45%) in perioperative complications (19.2% vs. 35%, p=0.008). There was no difference in rates of major complications, biliary, pulmonary or wound-related complications.No difference on the clearance of surgical margins between techniques was found. In fact, TLLR group showed larger resection margins than patients undergoing OLR (12.4±13.7 mm vs. 5.8±5.5 mm, p<0.001).

**TABLE 2:** Perioperative results before and after propensity score matching (PSM)

	TLLR (n=120)	OLRbefore PSM (n=470)	p	OLRafter PSM (n=120)	p
Blood loss (ml) Mean±sd Median (interquartile range)	553.8±553.8 225 (92-800)	777.9±890.2 500 (300-975)	0.004	680.7±663.5 500 (250-800)	0.055
Transfusion (%)	16 (13.3%)	83 (17.7%)	0.277	15 (12.5%)	0.853
Operative time (min) Mean±sd Median (interquartile range)	286.8±133.4 265 (180-375)	385±133.4 375 (290-465)	<0,001	352.4±141.5 315 (255-420)	<0.001
ICU (%)	91 (75.8%)	437 (93.2%)	<0.001	111 (92.5%)	<0.001
ICU stay (days) Mean ± sd Median (interquartile range)	1.9±1.2 1.5 (1-2.8)	2.7±2.3 2 (1-3)	<0,001	2.5±2.2 2 (1-3)	0.031
Hospital stay (days) Mean±sd Median (interquartile range)	5,8±3,9 5 (3-8)	9,9±8,9 9 (6-11)	<0,001	9,9±9,3 9 (7-10)	<0,001
Morbidity^a^ (%)	23 (19.2%)	164 (34.9%)	<0.001	42 (35%)	0.008
Major complications^a,b^ (%)	5 (4.2%)	50 (10.6%)	0.003	11 (9.2%)	0.194
Wound-related complications^a^ (%)	2 (1.7%)	16 (3.4%)	0,55	5 (4.2%)	0.446
Biliary complications^a^(%)	4 (3.3%)	16 (3.4%)	1	5 (4.2%)	1
Pulmonary complications^a^ (%)	4 (3.3%)	44 (9.3%)	0,037	10 (8.3%)	0.166
Mortality^a^ (%)	0	20 (4.3%)	0,006	3 (2.5%)	0.122
Margins (%) Free Compromised	118 (98.3%) 2 (1.7%)	433 (92.1%) 37 (7.9%)	**0,012**	114 (95%) 6 (5%)	0.281
Margin (mm) Mean±sd Median(interquartile range)	12.4±13.7 9 (5-15)	6.8±7.5 4 (2-10)	<0.001	5.8±5.5 4,5 (2-7.8)	<0.001

OLR=open liver resection;TLLR=total laparoscopic liver resection;sd=standard deviation; ICU=intensive care unit; ^a^up to 90 days after the surgical procedure; ^b^Dindo-Clavien III-IV

## DISCUSSION

Initial development of MILS was slow, withheld by many barriers^8^ ^,^ ^26^. The first limit to be overcome was the translation of open techniques to the laparoscopic approach such as liver mobilization, vascular control and parenchymal transection. Additionally, other paradigms needed to be broken such as the risk of massive bleeding, the theoretical increased risk of gas embolism secondary to pneumoperitoneum, and concerns about oncological outcomes^8^ ^,^ ^24^.

The first LLR were described at the beginning of the 1990s, and were basically wedge resections of peripheral lesions^22^. Subsequently, anatomic resections, such as left lateral sectionectomy and major hepatectomies were reported^2^ ^,^ ^10^. The good initial results achieved at the beginning of the 2000s showed that MILS are both feasible and safe.

Over the past decade an increasing number of studies have been published comparing perioperative results of LLR and OLR, confirming the safety and potential benefits of minimally invasive surgery. A recent systematic review, including 43 comparative studies, showed that LLR are associated with lower blood loss, shorter hospital stay and fewer perioperative complications^6^. However, most of the studies included were retrospective, and therefore liable to selection bias. 

For this reason, one of the main criticisms of studies showing the benefits of LLR is that the results can be influenced by the intrinsic bias of observational studies. High quality data from randomized trials is the best way to overcome this limitation; however, at present there are only two randomized controlled trials published, both addressed to the comparison of open and laparoscopic left lateral sectionectomy^12^ ^,^ ^29^. The first by Ding et al.^12^ was a single centre study including patients treated for hepatolithiasis. The second (ORANGE II Trial)^29^ was unable to randomise a sufficient number of patients over four years, and was interrupted with a small number of participants. This shows that, despite being the best scientific evidence for the evaluation of the results of LLR, randomized controlled trials are difficult to conduct in clinical practice. In this context, international registries and well-designed observational are the most appropriate ways to produce evidence supporting LLR.

Only recently observational studies with high methodological quality have been published^1^ ^,^ ^9^ ^,^ ^25^. Matching methods allows the comparison of groups with less risk of bias. In our study we observed that after PSM both groups were homogeneous regarding the main clinical and surgical characteristics. It is worth highlighting that diagnosis (benign vs. malignant), the presence of chronic liver disease and type of procedures carried out were similar between the groups. 

In a recent systematic review, Zhang et al.^30^ included 10 high quality observational studies, which compared OLR and laparoscopy in patients with colorectal liver metastasis, observing a 43% reduction in perioperative complications, a similar result to that found in our study. They also showed lower blood loss, lower rate of blood transfusions and shorter hospital stay despite longer operative time. Regarding the oncological results, there was no increase in compromised margins, with a similar five-year overall survival and disease-free rates between the groups.

In contrast with other authors^6^ ^,^ ^30^, a shorter operative time for patients submitted to laparoscopy was observed in our study. This finding can be explained by the increased experience with MILS; our programme started in 2005, and currently carries out over than 400 minimally invasive hepatectomies. This means that the learning curve has been overcome and surgical steps has been standardised for a variety of minimally invasive procedures, which entails a significant reduction in operative time^9^. Recent studies have also demonstrated shorter operative time in patients submitted to LLR, mainly those submitted to minor resections and left lateral sectionectomy^19^. Ciriaet al.^6^ analysed publications after 2010 and observed shorter operative time in patients submitted to minor LLR when compared to patients undergoing similar OLR.

Recent observational studies and meta-analyses found lower blood loss in the LLR group^6^ ^,^ ^9^ ^,^ ^19^. In our study, we found a marginal decrease in the estimated blood loss after PSM (553.8±553.8 vs. 680.7±663 min, p=0.055). Factors that may have influenced this reduction are the development of new energy devices for liver transection, the image magnification afforded by laparoscopy, the pneumoperitoneum and the widespread use of linear staplers for controlling glissonean pedicles and large vessels^1^ ^,^ ^24^ ^,^ ^26^.

The reduction of hospital stay is a frequent outcome attributed to minimally invasive surgery^1^ ^,^ ^6^ ^,^ ^30^. Consistently, we observed a reduction of almost four days in the laparoscopic group. This finding should be interpreted as consequence of less necessity for and ICU stay, lower blood loss and lower morbidity rate^7^ ^,^ ^9^ ^,^ ^19^.

The fear of inferior oncological results in patients undergoing LLR was not demonstrated by the available studies. The main concern was that the laparoscopic two-dimensional vision, and the loss of tactile sensation could have resulted in a higher frequency of compromised margins. However, like in our study, several authors found similar R0 resections when compared to OLR, some of them obtaining wider margins in laparoscopic group^6^ ^,^ ^30^.

The major limitation of this study was the observational design, which can produce unbalanced groups in their baseline characteristics. For this reason, our study was designed to minimize bias. Selection bias was reduced excluding cases in which OLR is typically employed, such as two-stage hepatectomies and hilar cholangiocarcinoma. We believe that the use of a PSM equalized the groups for the main clinical, epidemiological and surgical characteristics, which made our results reliable.

## CONCLUSION

TLLR is feasible andsafe, when compared with well-matched patients submitted to OLR, TLLR is associated with shorter operative time, shorter ICU and hospital stay, as well as significant reduction in perioperative complications.

## References

[1] Aldrighetti L, Guzzetti E, Pulitanò C, Cipriani F, Catena M, Paganelli M (2010). Case-matched analysis of totally laparoscopic versus open liver
resection for HCC short and middle term results. J SurgOncol.

[2] Azagra JS, Goergen M, Gilbart E, Jacobs D (1996). Laparoscopic anatomical (hepatic) left lateral
segmentectomy-technical aspects. Surg Endosc.

[3] Belgihiti J, Clavien PA, Gadzijev, Garden JO, Lau YW, Makuuchi M, Strong RW (2000). The Brisbane 2000 terminology of liver anatomy and
resections. HPB.

[4] Berardi G, Tomassini F, Troisi RI (2015). Comparison between minimally invasive and open living donor
hepatectomy A systematic review and meta-analysis. Liver Transpl.

[5] Buell JF, Cherqui D, Geller DA, O'Rourke N, Iannitti D, Dagher I (2009). The international position onlaparoscopicliversurgery The
Louisville Statement, 2008. Ann Surg.

[6] Ciria R, Cherqui D, Geller DA, Briceno J, Wakabayashi G (2016). Comparative Short-term Benefits of Laparoscopic Liver Resection
9000 Cases and Climbing. Ann Surg.

[7] Coelho FF, Bernardo WM, Kruger JAP, Jeismann VB, Fonseca GM, Macacari RL (2018). Laparoscopy-assisted versus open andpurelaparoscopic approach for
liverresectionand living donor hepatectomy a systematicreviewand
meta-analysis. HPB (Oxford).

[8] Coelho FF, Kruger JA, Fonseca GM, Araujo RL, Jeismann VB, Perini MV (2016). Laparoscopic liver resection Experience based
guidelines. World J Gastrointest Surg.

[9] Coelho FF, Kruger JAP, Jeismann VB, Fonseca GM, Makdissi FF, Ferreira LA (2017). Are Hybrid Liver Resections Truly Minimally Invasive A Propensity
Score Matching Analysis. J LaparoendoscAdvSurg Tech A.

[10] Di Fabio F, Samim M, Di Gioia P, Godeseth R, Pearce NW, Abu Hilal M (2014). Laparoscopic major hepatectomies clinical outcomes and
classification. World J Surg.

[11] Dindo D, Demartines N, Clavien PA (2004). Classification of surgical complications a new proposal with
evaluation in a cohort of 6336 patients and results of a
survey. Ann Surg.

[12] Ding G, Cai W, Qin M (2015). Pure Laparoscopic Versus Open Liver Resection in Treatment of
Hepatolithiasis Within the Left Lobes A Randomized Trial
Study. Surg Laparosc Endosc Percutan Tech.

[13] Fonseca GM, Jeismann VB, Kruger JAP, Coelho FF, Montagnini AL, Herman P (2018). Liver resection in Brazil a national survey. ABCD, Arq Bras Cir Dig.

[14] Giménez ME, Houghton EJ, Davrieux CF, Serra E, Pessaux P, Palermo M (2018). Percutaneous radiofrequency assisted liver partition with portal
vein embolization for staged hepatectomy (PRALPPS). ABCD, ArqBrasCirDig.

[15] Herman P, Krueger JAP, Perini MV, Coelho FF, Lupinacci RM (2013). Laparoscopic Hepatic Posterior Sectionectomy A Hand-assisted
Approach. Ann SurgOncol.

[16] Hirotugu A (1974). A new look at the statistical model
identification. IEEE Transactions on Automatic Control.

[17] Koch M, Garden OJ, Padbury R, Rahbari NN, Adam R, Capussotti L (2011). Bile leakage after hepatobiliary and pancreatic surgery a
definition and grading of severity by the International Study Group of Liver
Surgery. Surgery.

[18] Koffron AJ, Auffenberg G, Kung R, Abecassis M (2007). Evaluationof 300 minimallyinvasiveliverresectionsat a single
institution lessis more. Ann Surg.

[19] Macacari RL, Coelho FF, Bernardo WM, Kruger JAP, Jeismann VB, Fonseca GM (2018). Laparoscopic vs open left lateral sectionectomy: An update
meta-analysis of randomized and non-randomized controlled
trials. Int J Surg.

[20] Nitta H, Sasaki A, Otsuka Y, Tsuchiya M, Kaneko H, Wakabayashi G (2013). Impact of hybrid techniques on laparoscopic major
hepatectomies. J HepatobiliaryPancreat Sci.

[21] Nomi T, Fuks D, Kawaguchi Y, Mal F, Nakajima Y, Gayet B (2015). Learning curve for laparoscopic major hepatectomy. Br J Surg.

[22] Reich H, McGlynn F, DeCaprio J, Budin R (1991). Laparoscopic excision of benign liver lesions. Obstet Gynecol.

[23] Torres OJM, Linhares MM, Ramos EJB, Amaral PCG, Belotto M, Lucchese AM (2019). Liver resection for non-oriental hepatolithiasis. ABCD, ArqBrasCirDig.

[24] Tranchart H, O'Rourke N, Van Dam R, Gaillard M, Lainas P, Sugioka A (2015). Bleeding control during laparoscopic liver resection a review of
literature. J Hepatobiliary Pancreat Sci.

[25] Untereiner X, Cagnet A, Memeo R, De Blasi V, Tzedakis S, Piardi T (2016). Short-term and middle-term evaluation of laparoscopic
hepatectomies compared with open hepatectomies A propensity score matching
analysis. World J Gastrointest Surg.

[26] Vibert E, Perniceni T, Levard H, Denet C, Shahri NK, Gayet B (2006). Laparoscopic liver resection. Br J Surg.

[27] Von Elm E, Altman DG, Egger M, Pocock SJ, Gotzsche PC, Vandenbroucke JP (2008). The Strengthening the Reporting of Observational Studies in
Epidemiology (STROBE) statement guidelines for reporting observational
studies. J ClinEpidemiol.

[28] Wakabayashi G, Cherqui D, Geller DA, Buell JF, Kaneko H, Han HS (2015). Recommendations for laparoscopicliverresection a
reportfromthesecondinternational consensus conferenceheld in
Morioka. Ann Surg.

[29] Wong-Lun-Hing EM, van Dam RM, van Breukelen GJ, Tanis PJ, Ratti F, van Hillegersberg R (2017). Randomized clinical trial of open versus laparoscopic left
lateral hepatic sectionectomy within an enhanced recovery after surgery
programme (ORANGE II study). Br J Surg.

[30] Zhang XL, Liu RF, Zhang D, Zhang YS, Wang T (2017). Laparoscopic versus open liver resection for colorec-tal liver
metastases A systematic review and meta-analysis of studies with propensity
score-based analysis. Int J Surg.

